# Liver Imaging and Hepatobiliary Contrast Media

**DOI:** 10.1155/2018/2487405

**Published:** 2018-09-09

**Authors:** Catherine M. Pastor, Oliver Langer, Bernard E. Van Beers

**Affiliations:** ^1^Department of Radiology, Hôpitaux Universitaires de Genève, Geneva, Switzerland; ^2^Laboratory of Imaging Biomarkers, Centre of Research on Inflammation, UMR 1149, INSERM, University Paris Diderot, Paris, France; ^3^Austrian Institute of Technology, Vienna, Austria

In the past, great achievements have been made by analysing the drug plasma concentrations to understand their body distribution. At that time, the estimation of liver concentrations was not available. Therefore, when conducting pharmacokinetic studies, it was assumed that hepatocyte concentrations approximate plasma concentrations, with the drug equilibration across the sinusoidal membrane being obtained by passive diffusion. With the discovery of hepatocyte transporters that modify the transport rates across hepatocyte membranes, this assumption is no longer valid. The activity of sinusoidal transporters can be much higher than passive diffusion, increasing the hepatocyte concentrations over the plasma concentrations. Moreover, the drug concentrations generated by the hepatocyte uptake clearances are simultaneously modified by efflux clearances from hepatocytes into bile canaliculi and back into sinusoids. Thus, depending on the relative hepatocyte influx and efflux clearances, drug hepatocyte concentrations can exceed, equal, or be lower than plasma concentrations. Liver imaging can now estimate liver concentrations following the injection of hepatobiliary contrast agents and radiotracers. However, how these concentrations are created is partially unknown. For these reasons, we encouraged the submission of basic, translational, and clinical studies that increase the understanding of liver imaging with hepatobiliary contrast media.

We included six original articles and one review in the special issue. Two publications examined the pharmacokinetics of Gd-EOB-DTPA and Gd-BOPTA in rat livers. Myung-Won You and colleagues measure the Gd-EOB-DTPA vascular clearances in normal rats and rats with hepatectomy (70% and 90%). The serum Gd-EOB-DTPA concentrations identified the decreased uptake function during hepatectomy, and the authors propose this quantification as a new liver function test. A review written by Catherine Pastor shows the value of isolated and perfused rat livers to quantify the Gd-BOPTA distribution into liver compartments. In the experimental model, a gamma counter placed over rat livers detects over time the concentrations of radiotracers. The review summarises the effects of liver temperature, hepatic perfusion, and canalicular transporter deficiency on Gd-BOPTA hepatocyte concentrations. A clinical study by Lukas Haider and colleagues investigates whether the evolution of liver functions can be predicted in patients with advanced liver fibrosis after hepatitis C virus eradication. They show that liver MRI following the injection of Gd-EOB-DTPA may distinguish patients with high or low risk of liver decompensation. Such prediction might initiate an individualised surveillance strategy.

Four publications examine the pharmacokinetics of PET radiotracers in rodents. Marco Taddio and colleagues describe the pharmacokinetic modelling of new PET radiotracers in mice and evidence how transporter inhibition by cyclosporine decreases their liver clearance while increasing the renal clearance. In rats, Fabien Caillé and colleagues describe the labelling of metoclopramide and show that its transporter-mediated plasma clearance into hepatocytes is saturable and not modified by the P-glycoprotein inhibitor tariquidar. The P-glycoprotein canalicular transport of metoclopramide is inhibited by tariquidar which controls its liver exposure. Besides metabolism, this study highlights the role of hepatocyte transporters in the disposition of metoclopramide in rat livers. Given the increased interest in bile acid PET imaging to assess the transport function of livers, Stef De Lombaerde and colleagues explore *in vitro* the best ^18^F-labelled bile acids that are substrates of bile acid transporters. In mice, they show how these labelled bile acids can serve as PET biomarkers to analyse the hepatobiliary transport of bile acids. Andrea Testa and colleagues demonstrate the interactions of transporter inhibitors on the bile acid analogue [^18^F]LCATD transport across hepatocytes and suggest to use it in the early stage of drug development.

In summary, this special issue highlights the transport of several hepatobiliary contrast agents and radiotracers across hepatocytes. The transport across organic anion transporting polypeptide and multiple resistance-associated protein 2 is evidenced by the MR contrast agents Gd-BOPTA and Gd-EOB-DTPA. New labelled bile acids explore the transport across the Na^+^-dependent taurocholate cotransporting polypeptide and the canalicular bile salt export pump. Other transporters such as P-glycoprotein are involved in the hepatocyte transport of drugs. These transports are studied in normal livers and livers with chronic disease. The main relevant topics of the issue focus on the hepatocyte concentrations induced by these transport functions and the transporter-mediated drug-drug interactions. Readers interested by the research topic can find more information on the website of the Hepatocyte Transporter Network led by Catherine Pastor (https://www.unige.ch/hepatocyte-transporter-network/home/).

